# Have You Ever Seen a Robot? An Analysis of Children’s Drawings Between Technology and Science Fiction

**DOI:** 10.1007/s41979-023-00098-6

**Published:** 2023-05-01

**Authors:** Christian Giang, Loredana Addimando, Luca Botturi, Lucio Negrini, Alessandro Giusti, Alberto Piatti

**Affiliations:** 1 Laboratorio Media e MINT (MEM), Department of Teaching and Learning, University of Applied Science and Arts of Southern Switzerland, Locarno, Switzerland; 2 Dalle Molle Institute for Artificial Intelligence (IDSIA), USI-SUPSI, Lugano, Switzerland

**Keywords:** Digital literacy, Drawings, Educational robotics, Primary school, Secondary school

## Abstract

Technologies have become an essential part of the daily life of our children. Consequently, artifacts that imply the early adoption of abstract thinking affect the imagination of children and young people in relation to the world of technology, now much more than they did in the past. With the emerging importance of robots in many aspects of our everyday lives, the goal of this study is to investigate which mental representations children have about robots. To this end, drawings from 104 children aged between 7 and 12 years old were used as a map of representations, considering the drawings as a proxy capable of evoking learned or emerging mental frameworks. The drawings were analyzed in several steps: they were first labeled using binary descriptors and then classified using clustering methods based on Hamming distances between drawings. Finally, questionnaire items covering children’s perceptions about robots were analyzed for each of the resulting cluster separately to identify differences between them. The results show that there are relationships between the way children draw robots and their perception about robots’ capabilities as well as their aspirations to pursue a career in science. These findings can provide meaningful insights into how to design educational robots and learning activities for children to learn with and about robots.

## Introduction

With the digital transformation of modern societies, technology plays an increasingly important role in the lives of many people. This also concerns children, as digital technologies have equally become an integral part of their everyday lives in a technology-saturated environment (Vandewater et al., 2007). Many educational systems throughout the world have thus integrated digital competencies as core learning goals (Carretero et al., 2017; D-EDK, 2016; Guitert et al., 2017). In some cases, these are integrated under the broader umbrella of STEM disciplines (Science, Technology, Engineering and Mathematics; Li et al., 2020; Sullivan & Heffernan, 2016). As a result, schools and teachers have started to integrate digital technology into teaching and learning activities, and lesson plans have been developed for pupils to understand, live with, and safely and effectively use digital technologies. As a matter of fact, digitalization has become a top emphasis in education (see, e.g., European Commission, 2020; Keser et al., 2012).

Robots are part of this movement in two ways that can be labeled as learning *with* robots and learning *about* robots (Gaudiello & Zibetti, 2016): (a) on the one hand, educational robots are being used in class as instructional tools to support learning in different subject matters, especially STEM disciplines, as well as to teach soft skills such as collaboration (Khine, 2017; Nazikhovna, 2022; Papadakis & Kalogiannakis, 2022; Tselegkaridis & Sapounidis, 2022); (b) on the other hand, understanding robots and how they work is part of the content of “new” subjects such as informatics and technologies.

However, as it happens with most common artifacts, children do not first discover robots at school but carry with them dense and often unaware pre-instructional conceptions of robots. Children might have seen robots in media productions (e.g., films such as *Star Wars*, *Terminator*, or Wall*e), videogames (such as *Daemon Ex Machina* or *Steel Battalion*) or cartoons, adverts, and music clips; they might also have seen real robots, from domestic appliances such as robotic vacuum cleaners, kitchen tools, mars rovers, or industrial robotic arms.

Operational mental models can either emerge from direct experience or be transferred from either another person through vicarious experience, as well as from the media through mediated experience (Halford, 2014; Lee, 2004; Olson & Bruner, 1974). Children’s interactions with robots depend on their knowledge of the tool, the frequency of use, and the robot’s actual appearance and capabilities (Obaid et al., 2018) as well as its predicted capabilities (Bandura, 2021; Chu & Quek, 2014). Moreover, multiple elements may influence children’s operational mental models of robots, including their age, gender, media exposure, ICT exposure, and culture. As a result, it is critical to consider the aspects that culturally characterize diverse inquiry contexts, especially when the goal is to use robots as a learning tool. However, until today, this direction of research still appears to be under-explored.

To address this research gap, in this study, we aim at exploring children’s mental models of robots through their drawings. Investigating children’s perceptions through drawings has proven to be a viable way to understand their internal representations and has been applied to study children’s mental models of technologies such as computers (Mertala, 2019) or the internet (Botturi, 2021). In this work, we aim at expanding this approach to the field of robotics. Specifically, this study aims at addressing the following research questions through the analysis of children’s robot drawings:

- RQ1: What mental representations of robots do students in elementary and lower secondary school have?
- RQ2: Do these mental representations correspond to how they think about the capabilities of robots?
- RQ3: What connection can be drawn between these mental representations and their desire to pursue a career in science?

## Literature Review

### Robots as Instructional Tools: Learning with Robots

In recent years, interest in using robots as instructional tools has steadily increased. Initially, educational robotics has mainly been introduced with the aim to promote STEM disciplines. Researchers studying STEM education have extensively documented examples of how robots may be helpful and engaging instruments for learning that attempts to scientifically empower students (Chalmers, 2017; Mehrotra et al., 2021; Park & Han, 2016). Karim et al. (2015), for instance, investigated whether robots in the classroom may transform STEM teaching in K-12 and foster new approaches to learning, implying that the “use of robots in classroom has indeed moved from purely technology to education, to encompass new didactic fields”. Yet, they “identified several shortcomings, in terms of robotic platforms and teaching environments, that contribute to the limited presence of robotics in existing curricula, the lack of specific teacher training being likely pivotal” (p.1).

Robots are not only used for teaching STEM but also for other learning subjects, such as music (Han et al., 2009), foreign languages (Chang et al., 2010), and even ethical issues such as cyberbullying (Sanoubari et al., 2021). Furthermore, educational robotics promote transversal skills, such as problem-solving, computational thinking collaborative work, communication strategies, or creativity (Ardito et al., 2014; Chevalier et al., 2022; Negrini & Giang, 2019; Nelson, 2012; Park & Han, 2016). Robots have also been used to socially assist in the cognitive and intellectual development of children Mataric & J., 1999. The role of robots in such activities is primarily as a tool for learning (Giang et al., 2022). However, collaborative human-robot interactive learning and robot-based mentoring have also been studied (Mitnik et al., 2008). The results coincide with the conclusions of other research studies (Lee et al., 2013; Sullivan & Bers, 2015) showing the positive effects of introducing robotic resources to promote the development of skills and interests linked to the STEM knowledge areas.

In recent years, several works have investigated how to make learning with robots more effective. For instance, different models have been developed to improve the design of educational robotics tools and activities (Giang et al., 2019; Lauwers, 2010), to support teachers in preparing and evaluating educational robotics learning activities (Chevalier et al., 2021) and to integrate educational robotics as part of continuing professional development programs for teachers (El-Hamamsy et al., 2021). Nevertheless, the design and development of successful learning settings and activities also require knowledge about how children perceive and understand robots. For instance, understanding pupils’ conceptualization of robots can help teachers to avoid a disconfirmation experience by selecting more appropriate educational tools or robot kits (Storjak et al., 2022).

### Understanding Technologies: Learning About Robots

Learning *about* technologies (including, but not limited to, digital technologies) is part of many current school curricula, which frame the subject under different names, such as “Media and Informatics” (Switzerland), “Technology” (Italy), and “Technology and Design” (UK). How technology could or should be taught in school has no unique and agreed-upon answer. However, previous work has emphasized that “pupils should not only be taught to ‘know and understand’ but also to ‘create and do’” (Banks, 1994, p.2). Developing an approach to teaching technology boils down to finding a sound definition of what technology is. According to Naughton (1994), technology is not just artifacts or “things” but implies the definition of a *goal*, the activation of *knowledge*, and people working together in a *social organization*.

Understanding technologies today naturally comprises learning about robots, as they have become an integral part of our socio-technical society. If we consider them simply as artifacts, learning about robots entails identifying their key components (e.g., sensors, actuators, central unit) and functional processes (e.g., algorithms). If we extend the definition as suggested above, the real-life applications of robots and the social organizations which produce them and in which they are used also come into focus. As Flichy (2003) points out, we can neither conceive nor use a technique or technology without representing it: understanding robots means developing an operational mental model or concept of an artifact (Keil, 1989) as opposed to natural, social, and mental concepts (Carey, 1985). This can be interpreted as a progressive shift from a pre-scientific *everyday concept* to a scientific *mature concept* (van der Veer, 1994). For example, children of early age often resort to animistic or magic thinking (Lévi-Strauss, 1962) to explain complex experiences and objects such as computers (Mertala, 2019), to which they attach intelligence and will. Through experience and learning, the concept can then evolve to think of computers as “programmable machines,” a concept which is more interconnected with other concepts and supported by experience.

### Exploring Children’s Mental Models of Robots Through Their Drawings

Since the observation of children has become more rigorous and systematic over the past century, analyzing children’s drawings has been a rewarding field of investigation (Hargreaves, 1978; Rose et al., 2006). Anning and Ring (2004) expressed it as follows: “When a young child draws, they are offering us windows into their own developing understanding of the world and their relationships to significant people, things and places around them (…). However, what they draw and how they draw reflect the complexity of communication systems and visual images, signs and symbol systems in the domestic and leisure activities around them. They are encultured into using a wide range of graphicacy through their everyday experiences” (p. 2).

Science literacy, according to the Committee on Science Literacy and Public Perception of Science (Snow & Dibner, 2016), includes core literacies such as numeracy, textual literacy, visual literacy, and knowledge of graphs and charts. Science literacy is multidimensional in this sense since it incorporates “language, bodily gestures, mathematical symbols, and visual adjuncts” (Yore et al., 2003, p. 716) and is linked to other disciplines of study (Hand et al., 1999). The growing use of images (drawings, diagrams, photographs, and film) in science practices and science communication processes emphasizes the importance of the visual component in science, particularly for assessment practices (Bucchi & Saracino, 2016; Kress, 2003).

Bumby and Dautenhahn (1999) conducted one of the earliest studies analyzing children’s robot drawings. The authors held a series of design workshops with 38 children aged 7–11 years old, asking them to sketch a robot and then write a story about it. Finally, they were observed while engaging with various robots and sharing their opinions on robotics. Woods (2006) investigated children’s perceptions of robots’ looks, movement, and personality by asking children aged 9–11 to pick a robot picture and complete a questionnaire. The pictures revealed a variety of characteristics of the robots, including method of locomotion, body form, animal, human or machine look, presence or absence of face features, and gender. The questionnaire asked about the look and personality of the robots. The youngsters ranked the robots based on their emotional expression (from joyful to sad), as well as their behavioral purpose toward people (from friendly to aggressive). According to the authors, however, that categorization did not prove to be a suitable topology since, from the standpoint of scientific inquiry, each of the robot qualities could not explain the robot’s membership in one and only one category on its own.

In a study on robot design with children, Sciutti et al. (2014) discovered that attitudes regarding which elements are vital in a robotic companion shift with age. Children before the age of nine pay greater attention to the humanoid elements of the robot; older children, on the other hand, are more inclined to consider its abilities and functions. They also discovered that when children had previously seen and interacted with a robot, they pay greater attention to the robot’s perception and motor abilities rather than its appearance. This implies that children’s thinking abilities can be influenced by direct exposure to robots, such as in a robotics class. While robots should have certain human-like features to be easily readable by users, DiSalvo et al. (2002) also argued that robots should balance “humanness” and “robotness,” representing another valuable indicator when using robotics in the classroom. Tung (2016) found similar results, suggesting a moderate level of anthropomorphic appearance combined with appropriate social cues to enhance children’s acceptance of robots. Another study by Secim et al. (2021) analyzed 5–6 years old children’s robot drawings. Children were asked to draw a robot before and after a 6-week training with robots. The comparison of the two drawings revealed that children’s last robot drawings had more mechanical features than their first drawings and that children used mostly angry, confused, and unhappy expressions in their second robot drawings.

While many works emphasized the importance of both learning *with* and *about* robots, only a few works have studied the mental models of robots which children already carry within them before being exposed to educational robotics activities. Better understanding of these models could help educators, researchers, and developers to design tools and activities that are better targeted to the learning objectives related to educational robotics activities. Previous works have given insight into different facets of children’s perceptions of robots but lacked the ability to connect these results with implications related to the objectives of learning with and about robots. In this work, we investigate the relationship of children’s mental models of robots with two such objectives, namely their knowledge about robot capabilities to perform human tasks as well as the children’s aspirations to pursue a career in science. Moreover, in contrast to previous works, we asked the children to make two different drawings each addressing a different question, allowing us to analyze their mental models from different lenses. While we use the insights from previous work to cluster children’s mental models, we also introduce a novel cluster-based approach to group similar robot drawings based on Hamming distances between feature vectors, providing a more quantitative approach than previous work that was mostly based on qualitative analyses. We finally analyze children’s perception with respect to robot capabilities and aspirations to become scientists for each cluster separately.

## Methods

Data were collected in the framework of the research project *Introducing People to Research in Robotics through an Extended Peer Community in Southern Switzerland* funded by the Swiss National Research Foundation. The project aimed at fostering dialogue between scientists and society. For this purpose, students from participating public schools were exposed to educational robotics activities^1^ as part of their regular classroom activities during the school years 2018/2019 and 2019/2020. Specifically, the objective of these activities was to teach the students how to use and program the educational robot Thymio (Mobsya, Renens, Switzerland; Chevalier et al., 2016) to solve different types of tasks.

**Fig. 1:**
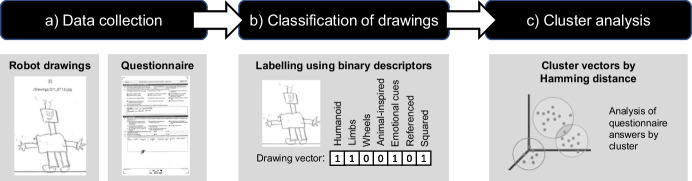
Schematic representation of the data collection (**a**), classification (**b**), and clustering process (**c**)

The data analyzed in this paper were collected at the outset of the project. At that moment, only a few pupils had already seen a Thymio robot in short workshops. During the first classroom session of the project, students had to complete a questionnaire, in which they were asked to draw robots as well as to express their opinion with respect to the use of robots and their aspirations of becoming scientists (Fig. 1a). The word “scientist” was used (instead of e.g., “engineer” or “roboticist”) as pupils in the target grade attend science classes that include topics from biology, chemistry, physics, and technology; on the other hand, they do not attend technology or engineering classes. Consequently, (a) they have a broad understanding of “science” as a set of disciplines connected to formal reasoning and technological applications and (b) they are more likely to have a richer imagination in connection to the science domain. The choice of the word “scientist” above other options for depicting science is also seen as effective for encouraging pupils to express their thoughts (Barman, 1997). The choice also provides a connection between this and previous research on scientist’s stereotypes (Buldu, 2006).

To analyze the children’s drawings, they were first manually labeled resulting in binary vectors for each drawing describing its characteristics (Fig. 1b). Clusters of similar drawings were then identified based on the Hamming distances between the vectors (Fig. 1c). Finally, it was analyzed how the answers to the remaining questions of the questionnaire differed across clusters. The following subsections present a more detailed description of each step of the process.

### Participants

A total of 106 pupils aged 7–12 years old participated in the data collection (Table 1). Specifically, the participating pupils were from five secondary school classes (the LSEC group, 60% of the sample), four primary school classes, and two special needs school classes from the Swiss Canton Ticino (the ELEM group, 40% of the sample) and from the Italian speaking part of Canton Grigioni. The convenience sample includes pupils that were participating in a larger research project and is thus not representative of the pupil population. Authorization for the participation of each pupil was granted by their legal guardians and the school directors as well as their class teachers. All data was collected anonymously.

**Table 1:** Demographic information of participating students

Group	Age	School level	No. of students
ELEM	7–9 years old	Elementary school	64
LSEC	11–12 years old	Lower secondary school	42

### Data Collection

Data were collected using a short questionnaire consisting of 4 items (Table 2) which was developed on purpose. After testing with some respondents in the target age groups and fine-tuning, the questionnaire was distributed on paper and was filled in using a pen or a pencil. For the first two items (Q1 and Q2), students had to provide their answers by making drawings of robots. The remaining items included questions about the children’s perception of the capabilities of robots (Q3) as well as their aspirations of becoming scientists (Q4).

**Table 2:** Items of the questionnaire distributed to the children

Item	Question	Answer type	RQ addressed
Q1	What picture comes to mind when you think of a robot? Draw it!	Drawing	RQ1
Q2	Think about a real robot you've seen before and try to draw it.	Drawing	RQ2
Q3	Do you think that one day the work of your dad or your mom can be done by a robot?	5-point Likert	RQ3
Q4	Would you like to become a scientist one day?	Binary choice	RQ3

### Classification of Drawings

**Fig. 2:**
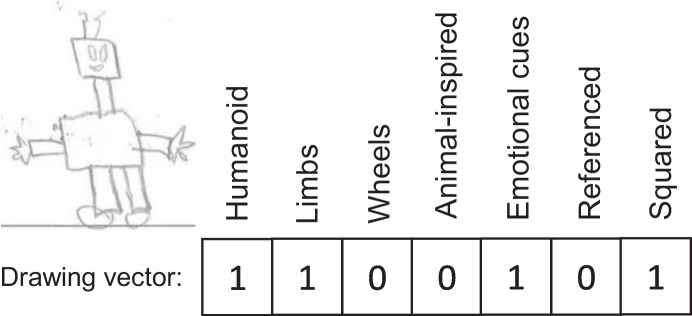
Example of a child’s robot drawing and its classification using the proposed binary descriptors

Children’s drawings from the first two items of the questionnaire (Q1 and Q2) were classified using descriptors based on the findings reported in recently published works about emotional cues (Rossi & Ruocco, 2019) and mental models of robots in younger children (age 5–6; Secim et al., 2021). Specifically, seven binary descriptors (each encoded by 1 or 0) were used to define whether certain characteristics of a robot were present in a drawing or not (Table 3). To illustrate the use of these descriptors, an example of a classified drawing is shown in Fig. 2. The descriptors were used by two evaluators to independently label a set of twenty drawings. The inter-rater reliability of the labeling process was computed using Cohen’s Kappa (*K*=0.87). Disagreements between evaluators were discussed, resulting in slight adjustments to the descriptors (i.e., fine-tuning the definitions). After that, each of the two evaluators labeled one-half of the remaining drawings using the same set of descriptors.

**Table 3:** Final list of binary descriptors for children’s robot drawings

Descriptor	Definition
Humanoid	The robot has a human-like shape, e.g., with a clearly identifiable head above the torso.
Limbs	The robot has arms or legs or both.
Wheeled	The robot has wheels for movement.
Animal-inspired	The robot’s shape resembles that of a recognizable animal.
Emotional Cues	The robot has eyes or mouth or both.
Referenced	Observing the drawing the researcher clearly recognizes one individual fictional or real robot s/he has seen already.
Squared	The drawing is mainly composed of straight lines.

### Cluster Analysis

Cluster analysis can be used to group samples and to develop ideas about the multivariate representations of the data set at hand. Due to the complex nature of data (drawings in a conditioned situation), cluster analysis results often strongly depend on the preparation of the data (e.g., choice of the transformation) and on the clustering algorithm selected. In this case, hierarchical functional clustering has been used to identify groups of drawings where the patterns in concentrations of characteristics are similar. Hierarchical clustering enables spatial covariance to be easily incorporated into the clusters by weighting the distance matrix by the covariance matrix (Hartigan, 1975).

The labeled vectors of the children’s drawings from the questionnaire items Q1 and Q2 were clustered in groups with similar characteristics by computing the Hamming distance between them. The Hamming distance was originally introduced in the context of error-correction codes, to detect the differences between two bitstrings (i.e., a sequence of ones and zeros) of the same dimension (Bookstein et al., 2002). Specifically, it aims at measuring the error introduced by noise over a message channel by comparing the number of positions with different values between the sent and the received message. As the drawing vectors used in this work all represent bitstrings with a length of seven, the Hamming distance was used as a measure to quantify the similarity between them. To form the clusters, the vectors that represent the most counts in the data set were selected as centroids. Specifically, we selected the three drawing vectors which were significantly more frequent than others as centroids: the vectors 1100101 (representing 27% of all vectors), 1100100 (19%), and 0000010 (6%). Subsequently, clusters were formed by assigning the remaining vectors to the centroid with the smallest Hamming distance. If a vector had an equal Hamming distance to multiple centroids, it was assigned to a cluster by manual inspection (3 occurrences). The rationale behind this path of analysis is that since the vectors have similar properties to the item being represented, their relationship may be explored (Fisher, 1996).

Pivoting on the cluster results, data from Q3 and Q4 was analyzed with descriptive statistics to investigate children’s professional development perspectives and expected robots’ impact on society.

## Results

### Think of a Robot: Drawings Based on Imagination

The results of the analyses of item Q1 of the questionnaire show that children’s drawings of robots based on imagination can mainly be classified into three clusters, which, for simplicity’s sake, we named after famous robots from cinematographic origins.

**Fig. 3:**
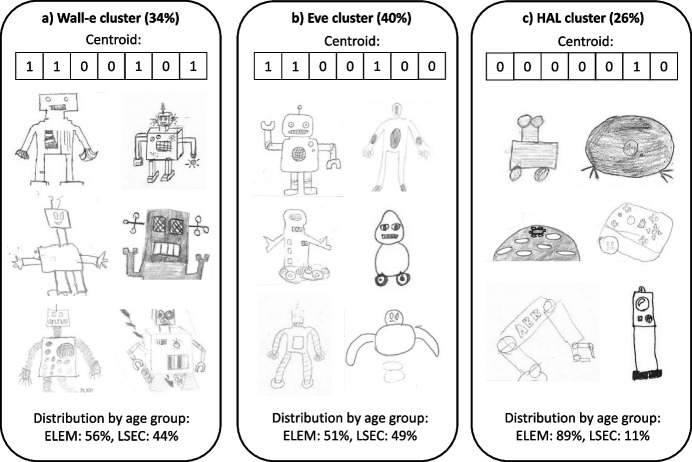
The three main clusters of drawing types identified from questionnaire item Q1. Each cluster was named after robots from cinematographic origins: Wall-e cluster (**a**), Eve cluster (**b**) and HAL cluster (**c**). For each cluster, the centroid vector, some example drawings, and the distribution by age group are presented

1. *Wall-e cluster*: The first cluster of drawings can be described as humanoid robots with predominantly squared shape (Fig. 3a, representing 34% of all answers). It contains 4 vectors, and its centroid was the drawing vector 1100101 which describes humanoid robots with limbs, emotional cues, and squared shape. Drawings of the centroid are the most common overall, representing 27% of all submitted drawings. As compared to the overall age group distribution of all participants (60% elementary and 40% lower secondary school), we found elementary school students to be slightly under-represented (56%) as compared to lower secondary school students (44%).
2. *Eve cluster:* The second group also consists of humanoid drawings with similar characteristics to the first group. However, a major difference is that the drawings in this group are not predominantly squared, but instead also include curved shapes (Fig. 3b, 40% of all answers). The cluster contains 9 vectors, and its centroid is the drawing vector 1100100 describing humanoid robots with limbs and emotional cues but drawn not only with straight lines. The centroid represented 19% of all submitted drawings. With only 51% of all drawings in this cluster, students from elementary school were under-represented as compared to lower secondary school students (49%).
3. *HAL cluster*: The last group of drawings represents non-humanoid robots such as industrial robotic arms, robotic vacuum cleaners, educational robots, or rovers (Fig. 3c, 26% of all answers). While they all have in common that they are non-humanoid robots, there is some variation with respect to the remaining descriptors: this cluster contains 15 vectors, and its centroid is the vector 0000010 (6% of all submitted drawings), which describes referenced non-humanoid robots. With 89% of all drawings in this cluster, students from elementary school were strongly over-represented as compared to lower secondary school students (11%).

### A Robot You Have Seen: Drawings Based on Real Robots

**Fig. 4:**
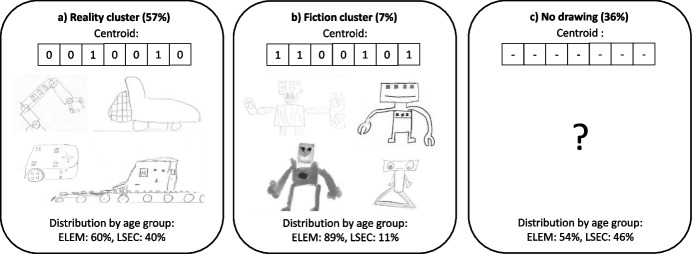
The two main clusters of drawing types identified from questionnaire item Q2 (reality cluster (**a**) and fiction cluster (**b**)) as well as the cluster that did not submit a drawing for this question (**c**). For each cluster, the centroid vector and some example drawings and the distribution by age group (ELEM, elementary school; LSEC, lower secondary school) are presented

The analysis of the children’s drawings from questionnaire Q2 reveals that when asking children about real robots they have seen before, three main clusters emerge:

1. *Reality cluster:* The first cluster, representing non-humanoid robots is dominant (Fig. 4a, representing 57% of all submitted drawings). It contains 15 drawing vectors; however, its centroid 0010010 is by far the most prevailing, representing 39% of all submitted drawings. The centroid of this group describes non-humanoid, wheeled robots. Moreover, since most of the robots in this cluster can be clearly associated with real, existing robots, we named it a *reality* cluster. With a distribution of 60% and 40%, respectively, we found that both elementary and lower secondary school students were represented proportionally to the number of participants in both groups.
2. Fiction cluster: The second, but by far less populated, group of drawings is humanoid robots (Fig. 4b, 7% of all submitted drawings). It consisted of 9 vectors with 1100101 being its centroid (1% of all submitted drawings). The centroid describes squared humanoid robots with limbs and emotional cues, possibly robots that children have seen in fictional products like movies or comics. For this reason, we named this group of drawings a *fiction* cluster. For this cluster, we found that elementary school students were strongly over-represented (89% of all drawings in this cluster), while lower secondary school students were strongly under-represented (11%).
3. *No drawing:* For this questionnaire item, there was also a large proportion of students that did not provide an answer (36%). Since for this question students were asked to draw robots that they had seen before, the high proportion of students not providing an answer indicates that more than a third of the participating students have never seen an artifact that they considered to be a robot. In terms of age group, elementary school students were slightly under-represented (54%) as compared to lower secondary school students (46%).

**Fig. 5:**
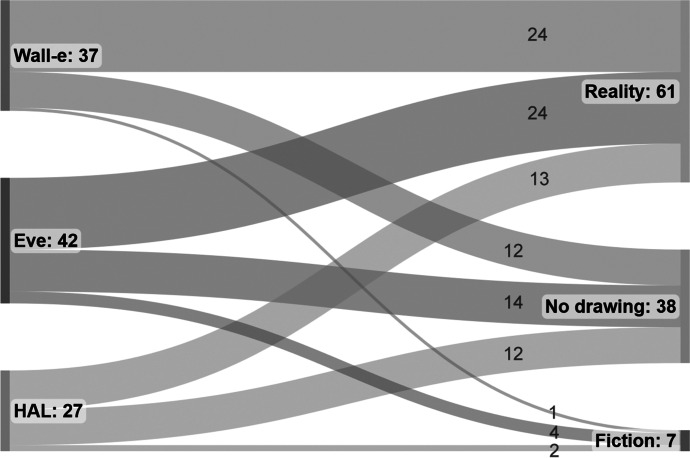
Sankey chart illustrating the relationships between the clusters from item Q1 (left) and the clusters from item Q2 (right). The labels indicate the absolute number of drawings in each cluster

We then analyzed the relationships between the clusters from Q1 and Q2 (Fig. 5). Students who made drawings in the *Wall-e* and *Eve* clusters for Q1 were most likely to draw robots in the *Reality* cluster for Q2. While this was also the case for the *HAL* cluster, almost as many students from this cluster did not provide an answer to this item. There were also students in the *Wall-e* and *Eve* clusters that did not provide answers to item Q2, but their proportion was lower. Making drawings in the *Fiction* cluster was the least likely for all three clusters in Q1.

### Questionnaire Answers by Cluster

We finally analyzed the children’s answers to the questionnaire items Q3 and Q4 for each of the clusters from Q1 and Q2 separately to investigate whether the way they draw robots is correlated with their personal perceptions about robots and a career in science.

**Fig. 6:**
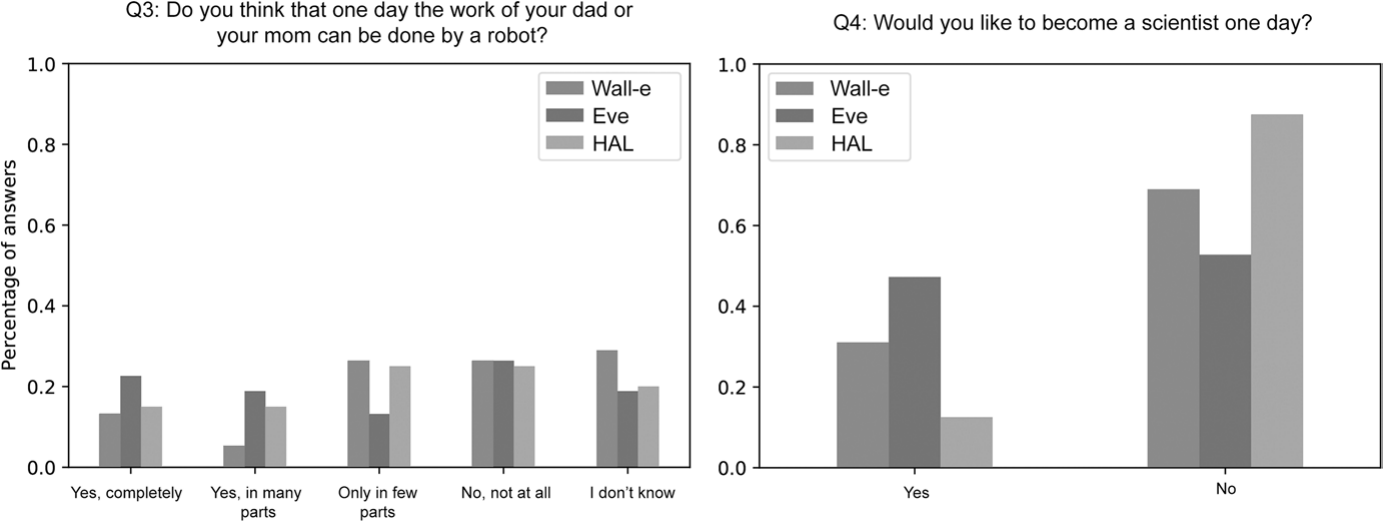
Answers to questionnaire items Q3 (left) and Q4 (right) by drawing clusters from Q1

When clustered by their drawings from item Q1 we found that children with drawings in the *Eve* cluster were more likely to believe that robots could one day perform the work of their parents than children in the other two clusters (Fig. 6 left). Moreover, there was a higher proportion of children in this cluster who indicated that they would like to become a scientist (Fig. 6 right). Children with drawings in the *HAL* cluster, on the other hand, appeared to be the least interested in becoming scientists. It is noteworthy that we also found a large proportion of students in all clusters that did not know how to answer Q3.

**Fig. 7:**
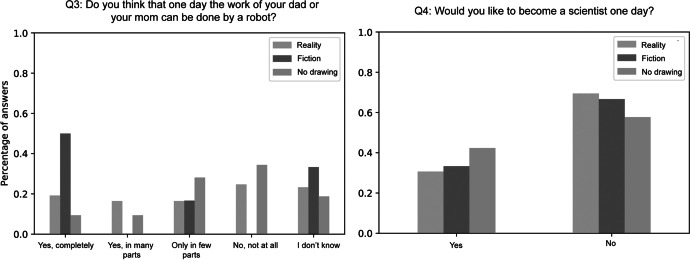
Answers to questionnaire items Q3 (left) and Q4 (right) by drawing clusters from Q1

When clustered by their drawings from item Q2, even larger differences emerged between clusters for item Q3. Children with drawings in the *Fiction* cluster were more convinced that robots could one day perform the work of their parents, while children from the *Reality* cluster were more skeptical (Fig. 7 left). As a matter of fact, more than 50% of the children in the *Fiction* cluster indicated that robots could completely perform the work of their parents. However, due to the very small sample in this cluster, it should be noted that these results have only limited significance. The students who were the most skeptical about robots’ capabilities were those who did not provide a drawing for Q2. With respect to the question if the students would like to become scientists, we did not observe strong differences in this case. With about two-thirds not wanting to become scientists, students had similar aspirations in this regard across clusters (Fig. 7 right).

## Discussion

In this work, we studied children’s mental models of robots by analyzing their drawings. In contrast to previous studies that used similar approaches (Bumby & Dautenhahn, 1999; Secim et al., 2021), in the present study, we asked the participating children to make two robot drawings, each addressing a different question. This allowed us to analyze the children’s mental models through different lenses as well as to study the relationships between the drawings.

With regard to our first research question RQ1 (“What mental representations of robots do students in elementary and lower secondary school have?”), we found that the children’s drawings of imaginary robots could be grouped into three clusters. The clusters represented a spectrum from predominantly human-shaped robot drawings (*Eve* cluster) to mechanical humanoid robots (*Wall-e* cluster) to purely mechanical robots (*HAL* cluster). As reported in the work of Sciutti et al. (2014), young children give more importance to human-like appearance, whereas older kids emphasize the robot’s ability to perform actions (e.g., moving, grasping, talking). Interestingly, the analysis of the age distributions in the three clusters showed that in our case, the opposite was observed. Compared to the secondary school students, the younger elementary school students appeared to be slightly under-represented in the two human-like clusters (*Wall-e* and *Eve*), and they by far dominated the mechanical cluster (*HAL*), putting a stronger emphasis on the robot’s capabilities. A possible explanation for this observation could be that among the participating children, a higher proportion of the younger children had already been exposed to educational robotics as part of their classroom activities. As a matter of fact, many of the drawings in the *HAL* cluster depicted the educational robot Thymio, which has already been introduced in some of the participating schools. This hypothesis aligns with the results of Secim et al. (2021), who found that children would draw robots with more mechanical features after being exposed to educational robotics activities, and confirms the importance and impact of educational robotics activities.

When analyzing the drawings of robots that they had seen before, we again found three main clusters of drawings. More than half of the children drew realistic robots (such as industry, domestic or educational robots), and both age groups were represented according to their proportion in the sample. Remarkably, more than one-third of the participants did not submit a drawing to this question, indicating that they have never seen an artifact that they considered to be a robot. This is particularly interesting, because except for autonomous vacuum cleaners and lawn mowers, none of the children drew domestic robots such as intelligent laundry machines, kitchen appliances, or other smart home devices. This indicates that additional efforts are needed to raise awareness about how ubiquitous intelligent devices function and that they can equally be considered “robots” in the broader sense. Finally, we also found a small cluster consisting of robot drawings that can be inspired by fiction. This, once more, emphasizes the importance of fiction in (especially younger) children’s mental models of robots. These findings illustrate that the framing of the question has a large impact on what type of robot children will draw. It also confirms our hypothesis that many children possess multiple mental models of robots, leading them to draw robots as both human-like and mechanical artifacts. However, we also observed that some children have a strictly fictional view of robots (those who drew *Wall-e* or *Eve* robots in the first drawing and a fictional or no robot for the second). As suggested by Belpaeme et al. (2013), our findings confirm that children have preconceptions of what robots can and cannot do.

We finally investigated the remaining two research questions RQ2 (“Do these mental representations correspond to how they think about the capabilities of robots?”) and RQ3 (“What connection can be drawn between these mental models and their desire to become scientists?”) by looking at the children’s answers to the questionnaire items for each cluster separately. For the first clusters of drawings, we found that children who drew the most human-like robots were more convinced than children from the other two clusters that robots could one day do the work of their parents. Whether this can be interpreted as a positive (robots to help the human workforce) or a negative perception (robots replacing the human workforce) cannot be conclusively determined based on the data available. Considering the second question, however, the perception could be interpreted as rather positive: children who drew human-like robots were more inclined to become scientists, followed by the ones who drew mechanical humanoid robots. Children drawing purely mechanical robots showed the least interest in becoming a scientist. Previous work has suggested that children show more positive closeness, enjoyment, and affect in the presence of robots than playing alone or with inanimate objects (Van Straten et al., 2020). It could be that more “mechanical” robots appear to be inanimate and hence less interesting as compared to robots with human-like traits. However, it should also be noted that for every cluster, more than half the children do not consider a career in science. Future work could build on these results to understand what motivates children to pursue a science career and how potentially interested children can be best supported to successfully reach this goal.

Extending the analysis to the second set of drawing clusters showed an even stronger trend for the first question. More than half of the children who drew robots that were inspired by fiction were convinced that robots could one day perform the work of their parents, whereas this was only the case for one-fifth of the children who drew realistic robots. Interestingly, children who did not submit a second drawing were the most skeptical. It is possible that these children are not aware of the capabilities of state-of-the-art robots since they have never seen them in real life. However, further work is needed to draw more substantial conclusions with regard to this question.

### Implications

This study clearly indicates that children have at least a naive conception of what a robot is and formulate expectations about how it should look and operate. Moreover, such conceptions are diverse. When introducing (or even designing) educational robots to (and for) pupils, considering such conceptions can help connect to the children’s experiences and consequently adjust their expectations avoiding both over-enthusiasm and disappointment. While robots should have certain human-like features to be easily readable by users, DiSalvo et al. (2002) also argued that robots should balance “humanness” and “robotness,” representing another valuable indicator when using robotics in the classroom. Our results seem to suggest that most pupils expect robots to share human features (being otherwise simply “machines”), while keeping, for half of our participants, the edgy features that make them different from us. In the light of our findings, education *about* robots acquires even more relevance: pupils’ imagination is densely populated with pictures and characters from the media, and consequently keener to view robotics in utopian or dystopian terms, than with realism. It is interesting to observe that many children drew different things and then asked about what they had in mind and what they had seen—as if half-aware that what they *think* is a robot is different from what they *know* it is. Educators, researchers, and developers should be aware of this varied perception among children and find ways to support all children to develop a holistic mental model of robots as part of our modern society. Working from this gap between knowledge and imagination can be a rewarding and motivating approach. While further work is needed to consolidate the findings of this study, some of the insights can already be leveraged in future research in this direction and integrated into educational robotics activities. Future work could extend this approach to greater and possibly randomized samples of participants, while considering features such as age, gender, culture, drawing skills, and exposure to media and ICT. Moreover, the descriptor vectors could be extended by additional features such as colors, displayed emotions, and allowing for more than just binary categories. This could enable the discovery of more complex and elaborated mental models through the analysis of drawings. For the same purpose, different framings of the drawing questions could be explored.

## Limitations

This study is exploratory in nature, and its results are hardly generalizable, because data were collected from a non-randomized sample that is not representative of the population. Analyzing the differences in the two drawings, we have observed that the prompt and the context influence what is represented. The drawings we examined were part of a larger survey, and as a result, they were subject to its limitations. For instance, students were only given a small amount of space on pre-printed pages, and they did not use colored pencils. It is reasonable to assume that the designs would have been different and potentially prompted distinct features in mental models under different circumstances. A variety of other methodological biases may be added to this study, also referring to the scientific literature on children’s sketching techniques. As opposed to allowing children to freely express themselves with their own conceptions of the concept of robotics, the choice was made to link scientists and robots. Similar issues include the incomparability of the groups considered in the research examined because of the pandemic period, the impossibility of determining the effects of any intervention variables a posteriori, the specificity of the educational situations considered, and so forth. Furthermore, some of the descriptors used in this study to classify the drawings were inspired by previous work that was conducted with younger children (Secim et al., 2021). It can be assumed that for the mental models of older children, such as those who participated in this study, other descriptors could yield more appropriate representations. Nonetheless, we believe that the results of this study shed some light on critical issues concerning both teaching *with* robots and teaching *about* robots. We believe that this type of exploration if embedded in the required broader long-term research effort to explore the actual benefits and limitations of educational robotics and of robotics in STEM education (Tselegkaridis & Sapounidis, 2022) would be beneficial to the further development of this field.

## Data Availability

The data collected in this study are not publicly available, as parts of them contain personal information. The data are, however, available from the authors upon reasonable request and with the permission of the schools and parents of the participating students.

## Footnotes

1 The materials can be found on the platform www.roteco.ch
